# Complications of Free Flap Reconstruction in Maxillary and Mandibular Defects: A Systematic Review and Meta-Analysis

**DOI:** 10.3390/jcm15020797

**Published:** 2026-01-19

**Authors:** Fabio Maglitto, Stefania Troise, Federica Calabria, Serena Trotta, Giovanni Salzano, Luigi Angelo Vaira, Vincenzo Abbate, Paola Bonavolontà, Giovanni Dell’Aversana Orabona

**Affiliations:** 1 Maxillofacial Surgery Unit, Department of Neurosciences, Reproductive and Odontostomatological Sciences, University of Naples Federico II, 80131 Naples, Italy; fabio.maglitto@aoufedericoii.unina.it (F.M.); stefania.troise@unina.it (S.T.); serenatrotta99@icloud.com (S.T.); giovannisalzanomd@gmail.com (G.S.); vincenzo.abbate@unina.it (V.A.); paola.bonavolonta@unina.it (P.B.); giovanni.dellaversanaorabona@unina.it (G.D.O.); 2 Maxillofacial Surgery Unit, University Hospital of Sassari, 07100 Sassari, Italy; lavaira@uniss.it

**Keywords:** mandibular reconstruction, maxillary reconstruction, microsurgery reconstruction, osseous free flap, postoperative complications

## Abstract

**Background:** Microvascular osseous free flaps play a central role in head and neck reconstruction; surgeons often rely on fragmented and inconsistently reported data when counselling patients and planning reconstructive strategies. This systematic review and meta-analysis aimed to quantify postoperative complication rates and to evaluate complication patterns according to flap type. **Methods:** The study protocol was registered in PROSPERO (CRD420251237516). Studies published between January 2000 and November 2025 reporting postoperative complications following mandibular or maxillary reconstruction with osseous free flaps were identified. Eligible studies included adult cohorts with a minimum sample size of twenty patients. Random-effects meta-analyses of proportions were conducted. Risk of bias was assessed using the ROBINS-I tool. **Results:** Fourteen retrospective studies encompassing 1198 flaps were included. The pooled incidence of total flap loss was 6% (95% CI 3–9%), and partial flap loss was 6% (95% CI 3–10%). The pooled rates for postoperative infection, fistula formation, and wound dehiscence were 7% (95% CI 2–22%), 12% (95% CI 7–20%), and 16% (95% CI 8–31%), respectively, with substantial heterogeneity. Fibular free flaps demonstrated pooled rates of 6.1% for total flap loss, 6.6% for partial flap loss, 9.0% for infection, 10.4% for fistula formation, and 17.1% for wound dehiscence. For scapular free flaps, pooled total flap loss was 5% (95% CI 1–29%). DCIA flaps demonstrated hardware-related complications (8.1%), fistulas (16.7%), bone exposure (4.2%), and wound dehiscence (29.7%). Donor site morbidity was inconsistently reported and could not be quantitatively synthesized. **Conclusions:** Osseous free flap reconstruction shows relevant complication rates, highlighting the need for standardized reporting to support evidence-based decision-making.

## 1. Introduction

Reconstruction of segmental defects of the mandible and maxilla represents one of the most demanding challenges in maxillofacial surgery [1]. These defects commonly arise from oncologic resection, trauma, osteonecrosis, or osteoradionecrosis, and advanced infections, often resulting in substantial functional and aesthetic impairment [2,3,4]. Microvascular osseous free flaps have become the gold standard for the reconstruction of mandibular and maxillary continuity in contemporary head and neck reconstructive surgery, allowing reliable restoration of mastication; speech; facial contour; and, when indicated, implant-supported dental rehabilitation [5,6]. Among the available donor sites, fibula, scapula, and iliac crest osteocutaneous flaps remain the most widely adopted options, each with unique anatomical characteristics and reconstructive advantages [7,8,9].

Despite their widespread use and high success rates, osseous free flap reconstructions are associated with a broad spectrum of postoperative complications [10,11]. Reported rates of total or partial flap loss, infection, wound dehiscence, fistulas, donor site morbidity, and hardware-related complications vary considerably across published studies, with several outcomes, particularly donor site morbidity, being inconsistently defined and variably reported. This variability reflects differences in flap selection, patient comorbidities, etiology, surgical technique, perioperative management, and follow-up. Additionally, heterogeneity in definitions and reporting standards complicates direct comparison between studies and limits the ability to derive accurate estimates for clinical counselling and risk stratification [12,13,14].

Current evidence is largely composed of single-center retrospective series or studies focused on specific flap types, leading to a fragmented understanding of complication patterns in maxillofacial reconstruction [13,15,16]. To the best of our knowledge, a comprehensive and standardized synthesis of postoperative complications across different osseous free flap types and reconstructive sites, encompassing both mandibular and maxillary reconstruction, remains lacking [17]. Given the growing emphasis on outcome predictability, perioperative optimization, and long-term functional rehabilitation, clarifying complication rates is essential for improving patient counselling, redefining surgical decision-making, and guiding future reconstructive strategies.

The primary objective of this systematic review and meta-analysis is to quantify and summarize the overall incidence of postoperative complications following osseous free flap reconstruction of mandibular and maxillary defects.

A secondary objective is to estimate complication rates according to flap type by performing proportional meta-analyses for the most commonly used osseous free flaps (e.g., fibula, scapula, and iliac crest) when sufficient data were available. The null hypothesis is that no differences exist in postoperative complication rates among different osseous free flap types, but this review aims to show that identifying accurate incidence and complication rates for these flaps allows surgeons to optimize surgical planning by selecting the most suitable patients and the most appropriate flap for each case, thereby minimizing predictable complications.

## 2. Materials and Methods

### 2.1. Study Protocol

This systematic review and meta-analysis was conducted in accordance with the Preferred Reporting Items for Systematic Reviews and Meta-Analyses (PRISMA 2020) guidelines [18]. The study protocol was prospectively registered in the International Prospective Register of Systematic Reviews (PROSPERO; registration number CRD420251237516). The protocol predefined the research question, eligibility criteria, search strategy, outcomes of interest, and methods for data synthesis, ensuring methodological transparency and minimizing the risk of reporting bias. Minor protocol amendments were made after registration; these did not affect the eligibility criteria, outcomes of interest, or planned data synthesis and are publicly available in the PROSPERO record. All stages of the review process were performed independently by two reviewers, and any disagreements were resolved by consultation with a third reviewer. To conduct this review, the authors consulted the available literature, and to the best of their knowledge, no systematic reviews and meta-analyses specifically analyzing the complications of all types of bone flaps have been recently published. For transparency, gray literature was not included, and only peer-reviewed sources were considered.

### 2.2. Search Strategy and Eligibility Criteria

The studies search was performed in PubMed/MEDLINE, Embase, Scopus, and Web of Science to identify studies published between 1 January 2000 and 24 November 2025 that reported postoperative complications following osseous free flap reconstruction of mandibular or maxillary defects. The search strategy was adapted for each database and relied on title-specific combinations of terms related to free flaps, hard-tissue reconstruction, and complications. The exact search strings were as follows: PubMed: (“free flap” [Title] OR “free flaps” [Title]) AND (“hard tissue” [Title] OR “hard tissues” [Title] OR “hard tissue defect” [Title] OR “hard tissue defects” [Title] OR osseous [Title] OR bony [Title] OR mandibular [Title] OR maxillary [Title]) AND (complication [Title] OR complications [Title]); Embase: (‘free flap’:ti [Title] OR ‘free flaps’: [Title]) AND (‘hard tissue’: [Title] OR ‘hard tissues’: [Title] OR ‘hard tissue defect’: [Title] OR ‘hard tissue defects’: [Title] OR osseous: [Title] OR bony: [Title] OR mandibular: [Title] OR maxillary: [Title]) AND (complication: [Title] OR complications: [Title]); Scopus: TITLE (“free flap” OR “free flaps”) AND TITLE (“hard tissue” OR “hard tissues” OR “hard tissue defect” OR “hard tissue defects” OR osseous OR bony OR mandibular OR maxillary) AND TITLE (complication OR complications); Web of Science: TITLE = (“free flap” OR “free flaps”) AND TITLE = (“hard tissue” OR “hard tissues” OR “hard tissue defect” OR “hard tissue defects” OR osseous OR bony OR mandibular OR maxillary) AND TITLE = (complication OR complications). A title-based search strategy was intentionally adopted to maximize topic specificity and to minimize the inclusion of studies primarily focused on soft-tissue reconstruction or unrelated microsurgical applications. The screening protocol was the following: start by eliminating duplicates and articles not in English. Then, articles were screened for irrelevant titles, pediatric populations, and time range. Among eligible articles, only observational studies were considered and, in particular, manuscripts explicitly describing defect site complications stratified by flap type. In particular, studies were considered eligible if they included adult patients undergoing mandibular or maxillary reconstruction using osseous or osteocutaneous microvascular free flaps and reported postoperative complications. Eligible study designs included prospective or retrospective cohort studies, comparative observational studies, and case series comprising at least twenty patients. Exclusion criteria were pediatric populations, soft-tissue-only flaps, non-microvascular reconstruction techniques, mixed cohorts without extractable osseous flap data, or absence of reported postoperative complications. Non-original articles, single case reports, very small case series, technical notes, and conference abstracts lacking analyzable data were excluded. In addition to database searching, a manual search of all the bibliographic entries of the articles included in the review was conducted, so as not to leave out interesting works. Similarly, from the manual search, only the articles that respected the aforementioned parameters were considered.

### 2.3. Data Collection Process

All records retrieved from the database searches were imported into the EndNote Web Clarivate Analytics (https://access.clarivate.com/login?app=endnote (accessed on 14 December 2025)), where duplicates were automatically removed. The remaining studies were screened according to the predefined time frame (2000–2025) and assessed for relevance to osseous free flap reconstruction of maxillary and mandibular defects.

Two reviewers independently screened titles and abstracts, excluding studies clearly unrelated to the topic or not reporting postoperative complications. Articles considered potentially eligible underwent full-text evaluation, again independently and in accordance with the procedures outlined in the PROSPERO protocol. Any disagreements were resolved by consultation with a third reviewer.

Data from the included studies were extracted using a standardized form and comprised study characteristics, patient demographics, flap type, defect etiology, postoperative complications, donor site morbidity, and follow-up duration. Data extraction was performed independently by two reviewers, and only information explicitly reported in the articles was used; study authors were not contacted for additional data.

### 2.4. Outcomes and Definitions

The primary outcome of interest was the overall incidence of postoperative complications following osseous free flap reconstruction of maxillary and mandibular defects. The complications of interest of the defect site included total or partial flap failure, surgical site infection, wound dehiscence, orocutaneous or oronasal fistula formation, hematoma or seroma, bone-related complications, hardware-related complications, and donor site morbidity, when reported. All outcomes were extracted and analyzed according to the definitions provided by the original studies as standardized outcome definitions were not consistently available across the literature. When definitions slightly differed across studies, outcomes were grouped under common clinical categories based on the terminology used by the original authors. Secondary outcomes included differences in complication patterns according to the type of used osseous free flap, such as fibular, scapular-based, and iliac crest flaps. Data on reoperations, hardware-related complications, and follow-up duration were also extracted when explicitly reported. Analyses were performed on a per-flap basis when clearly specified; when this information was not explicitly stated, outcomes were analyzed as reported by the original authors. As not all studies reported all complication types, percentages were calculated as the number of events divided by the total number of flaps in which that specific complication was explicitly assessed and reported. Given the limited number of studies reporting comparable outcomes, the variability in denominators, and the absence of standardized definitions, a quantitative analysis of donor site complications was not considered methodologically appropriate. These outcomes were therefore summarized descriptively in the discussion.

No assumptions were made regarding missing or unreported data, and studies not reporting a specific outcome were not treated as having zero events. Owing to incomplete and heterogeneous outcome reporting, formal sensitivity analyses were not feasible.

### 2.5. Risk of Bias Assessment

Risk of bias was subsequently assessed using the ROBINS-I tool [19], following the same independent assessment process and discrepancy resolution strategy. In accordance with the registered protocol, reporting bias was evaluated qualitatively, and no certainty-of-evidence assessment was undertaken.

### 2.6. Statistical Analysis

Quantitative synthesis was performed using meta-analyses of proportions to estimate the pooled incidence of postoperative complications following osseous free flap reconstruction. Meta-analyses were conducted in R using RStudio (version 2025.09.2+418; Posit Software, PBC) and the *meta* package (version 8.2.1; R Foundation for Statistical Computing, Vienna, Austria). For each outcome, pooled estimates and corresponding 95% confidence intervals were calculated using a random-effects model implemented as a random-intercept logistic regression with logit transformation. Between-study heterogeneity (τ^2^) was estimated using a maximum-likelihood estimator, and random-effects confidence intervals were computed using a t-distribution-based approach. Statistical heterogeneity was assessed using the I^2^ statistic derived from Cochran’s Q. Meta-analyses were conducted for reconstruction site complications when at least five studies reported quantitative data for the same outcome. Studies not reporting a given outcome were treated as missing data and were excluded from the corresponding pooled analyses.

To explore potential differences in complication patterns according to flap type, flap-specific proportional meta-analyses were conducted for fibular free flaps. For scapular and iliac crest (DCIA) free flaps, quantitative synthesis was limited to outcomes with an adequate number of studies; when data were sparse or inconsistently reported, results were summarized using narrative synthesis. Direct comparative meta-analyses between different flap types were not performed, due to the limited number of comparative studies and the heterogeneity of outcome definitions. Publication bias assessment and meta-regression analyses were not conducted, as the number of studies available for individual outcomes was limited.

## 3. Results

### 3.1. Study Selection

The initial database search across Scopus, Embase, Web of Science, and PubMed/MEDLINE yielded 68 records. After removal of 35 duplicates, 33 unique records underwent title and abstract screening. Of these, 14 studies were excluded because they were not pertinent to the topic (*n* = 11), fell outside the predefined temporal range (*n* = 1), involved a pediatric population (*n* = 1), or were not published in English (*n* = 1). A total of 19 full-text articles were then assessed for eligibility. During full-text review, 11 articles were excluded for the following reasons: case reports (*n* = 6), conference abstracts (*n* = 2), reviews or meta-analyses (*n* = 2), and mixed cohorts without extractable osseous flap–specific data (*n* = 1).

**Figure 1:**
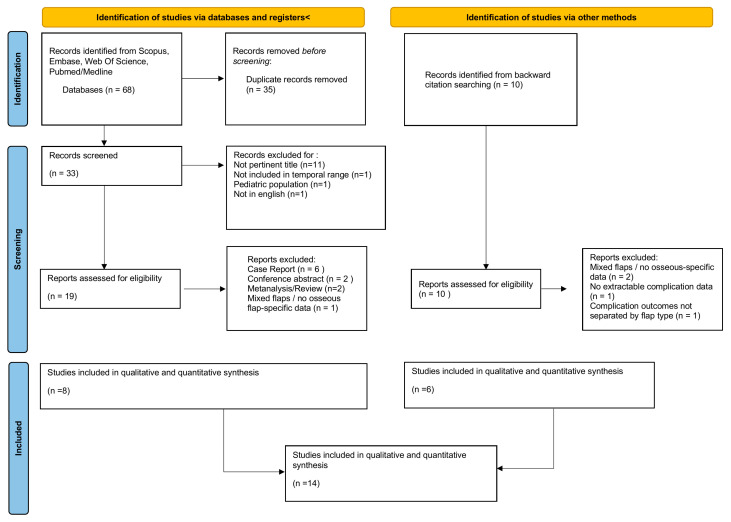
PRISMA flow diagram illustrating the study selection process according to PRISMA 2020 guidelines, including identification, screening, eligibility, and inclusion of studies for the qualitative and quantitative synthesis.

In addition to database searching, 10 additional records were identified through a manual research of backward citations. After full-text evaluation, four of these were excluded due to mixed flap populations without osseous-specific outcomes (*n* = 2), absence of extractable complication data (*n* = 1), or lack of complication reporting stratified by flap type (*n* = 1). Ultimately, 14 studies met all eligibility criteria and were included in the qualitative and quantitative synthesis [20,21,22,23,24,25,26,27,28,29,30,31,32,33]. The PRISMA flow diagram is illustrated in Figure 1. The PRISMA 2020 Main Checklist is available in the Supplementary Materials.

### 3.2. Study Characteristics

All data extracted from the 14 studies included in the review are summarized in Table 1.

The 14 studies included in this review were published between 2000 and 2025 and originated from ten different countries. Germany contributed three studies (21.4%) [26,27,33] followed by Spain with two studies (14.3%) [31,32]. Each of the remaining countries—France [20], Sweden [21], Korea [22], the Netherlands [23], India [24], Brazil [25], Belgium [28], Canada [29], and Japan [30]—contributed one study each (7.1%). All included studies adopted a retrospective design. When studies also included patients who underwent reconstructive procedures using techniques other than microvascular osseous free flaps, those patients were not included in the total count. Across the included studies, a total of 1173 patients underwent osseous free flap reconstruction of the mandible or maxilla, with 1198 flaps used overall, accounting for cases in which revision surgery required additional flap reconstruction. Sample sizes varied widely, ranging from small single-center cohorts of 25–43 patients [20,25] to large retrospective datasets exceeding 200 patients [24]. Sex distribution was reported in 12 of the 14 studies, accounting for 927 patients. Among these, 631 were male, corresponding to an overall male proportion of 68.1%. Age reporting was available in 10 studies, with mean patient age ranging from 43 to 62 years and an aggregated average of approximately 53.6 years. Follow-up duration was variably reported across the included studies. When specified, follow-up ranged from early postoperative assessments to long-term retrospective series extending up to 17 years. Several studies reported median or mean follow-up durations ranging approximately from 20 to 49 months, while others provided only minimum follow-up thresholds, time ranges, or qualitative descriptors. In some studies, follow-up duration was not explicitly stated. Most defects were post-oncological resection. Ten studies reported malignant disease as the primary indication for reconstruction [20,21,22,23,27,28,29,30,31,33]. Four studies described mixed etiologies, including combinations of malignant tumors, benign lesions, osteomyelitis, osteoradionecrosis, MRONJ, or trauma [24,25,26,27]. One study provided explicit counts (19 malignant and 7 benign) [32]. Most studies did not provide percentage distributions of etiology, limiting quantitative comparison across datasets. Mandibular defects were present in all included studies. Eleven studies reported exclusively mandibular or oromandibular reconstructions [20,23,24,25,27,28,29,30,31,32,33]. Three studies also included maxillary defects [21,26]. No study reported isolated maxillary defects. Fibular free flaps were the most commonly used osseous flaps, accounting for 996 reconstructions across the included studies and being reported in 13 of 14 publications [20,22,23,24,25,26,27,28,29,30,31,32,33]. Scapular-based flaps (SOFF, LSBF, and STFF) were described in 165 cases across five studies [21,22,26,29,30]. Deep circumflex iliac artery flaps were less frequent, totaling 37 reconstructions in two studies [26,27]. Donor site complications were inconsistently reported across the included studies, with substantial heterogeneity in outcome definitions, reporting methods, and level of detail. Several studies provided only qualitative descriptions or aggregate complication rates without extractable numerical data, while others explicitly excluded donor site morbidity from their analyses.

### 3.3. Reconstruction Site Complications

The complications reported for each flap are presented below. The number of cases was calculated based on the total number of flaps reported in the studies that explicitly assessed and reported that specific complication.

#### 3.3.1. Fibular Free Flap

Across the included studies, a total of 996 fibular free flaps were analyzed for postoperative complications. Total flap loss occurred in 50/765 cases (6.5%) [20,22,24,25,27,28,29,31,32,33]. Partial flap loss, mainly related to cutaneous or skin-paddle necrosis, was observed in 41/605 cases (6.8%) [20,22,23,25,27,28,29,30,32,33]. Reconstruction site wound infections were documented in 41/406 cases (10.1%) [22,23,28,30,31,33]. Fistula formation represented one of the most frequent complications, occurring in 107/679 cases (15.7%) [20,22,23,24,25,27,30,31]. Wound dehiscence was the most commonly reported complication, observed in 194/869 cases (22.3%) [20,22,23,24,26,27,28,30,31,33]. Bone-related complications, specifically intraoral bone exposure, were reported in 75/524 cases (14.3%) [23,24,26,27]. Hardware-related complications, including plate exposure, screw loosening, fixation failure, or plate fracture, were documented in 97/781 cases (12.4%) [22,23,24,25,26,27,29,31]. Seroma formation was reported in only two studies, occurring in 6/199 cases (3%) [22,23], while chyle leak was documented in 3/86 cases (3.5%), all reported by Lodders et al. Hematoma, described in only four studies, occurred in 9/267 cases (3.4%) [20,22,23,31]. Revision surgery, defined as return to the operating room for flap- or reconstruction-related complications, was required in 40/239 cases (16.7%) [23,24,31,32].

#### 3.3.2. Scapular Free Flap

Across the included studies, a total of 165 scapular-based free flaps were analyzed, including scapular osseous free flaps, lateral scapular border flaps, and scapular tip flaps. Total flap loss occurred in 13/157 cases (8.3%) [21,22,29,30]. Partial flap loss was uncommon, occurring in 1/73 case (1.4%) [29,30]. Reconstruction site wound infection was documented in 3/102 cases (2.9%) [21,22,30]. Fistula formation was observed in 13/102 cases (12.8%) [21,22,30]. Wound dehiscence was infrequently reported, occurring in 2/123 cases (1.6%), both described by Fujiki et al. [22,26,29,30]. Bone exposure was not explicitly reported in the included scapular flap series. Hardware-related complications, including plate exposure or fixation failure, occurred in 9/105 cases (8.6%) [22,26,29]. Hematoma was reported in 1/60 cases (1.7%), while seroma formation occurred in 2/60 cases (3.3%) [22,30]. Revision surgery was required in 5/73 cases (6.8%) [29,30].

#### 3.3.3. Deep Circumflex Iliac Artery (DCIA) Free Flaps

Across the included studies, a total of 37 deep circumflex iliac artery (DCIA) free flaps were analyzed [26,27]. No cases of wound infection, seroma, or hematoma were explicitly reported in the included DCIA studies. Hardware-related complications, including fixation or plate-related issues, were documented in 3/37 cases (8.1%). Fistula and bone exposure were documented in 4/24 cases (16.7%) and 1/24 cases (4.2%), respectively, all reported by Ritschl et al. [27] Wound dehiscence represented the most frequent recipient site complication, occurring in 11/37 cases (29.7%). No cases of total or partial flap loss or revision surgery were reported by Rendenbach et al. [1]; similarly, Ritschl et al. [27] explicitly stated that no such events were observed.

### 3.4. Risk of Bias Within Studies

**Figure 2:**
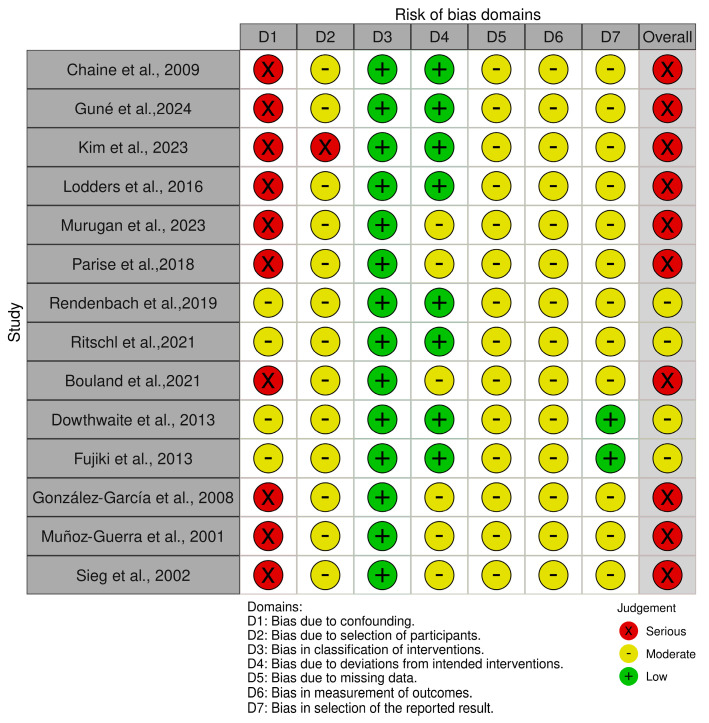
Risk of bias assessment of the included studies evaluated using the ROBINS-I tool across the predefined domains. Each study is classified according to the overall risk of bias derived from domain-level judgments [20,21,22,23,24,25,26,27,28,29,30,31,32,33].

Risk of bias was assessed for all included studies using the ROBINS-I tool [19]. Overall, the methodological quality of the available evidence was limited by the observational and retrospective design of all included studies. Most studies were judged to be at serious risk of bias overall, primarily driven by bias due to confounding (Domain 1), which was consistently rated as serious across the majority of studies. Across domains, bias due to classification of interventions (Domain 3) and deviations from intended interventions (Domain 4) was generally judged as low, reflecting clear reporting of reconstructive techniques and surgical procedures. Bias related to missing data (Domain 5), measurement of outcomes (Domain 6), and selection of the reported results (Domain 7) was most frequently assessed as moderate, mainly due to incomplete reporting and lack of standardized outcome definitions. Only a limited number of studies achieved an overall moderate risk of bias, whereas none were classified as low risk across all ROBINS-I domains. A detailed overview of domain-specific and overall risk of bias judgments is presented in Figure 2.

### 3.5. Quantitative Meta-Analysis: Overall Complications

Fourteen studies were included overall; however, the number of studies contributing to each meta-analysis varied according to outcome availability and completeness of data.

#### 3.5.1. Overall Flap Loss

**Figure 3:**
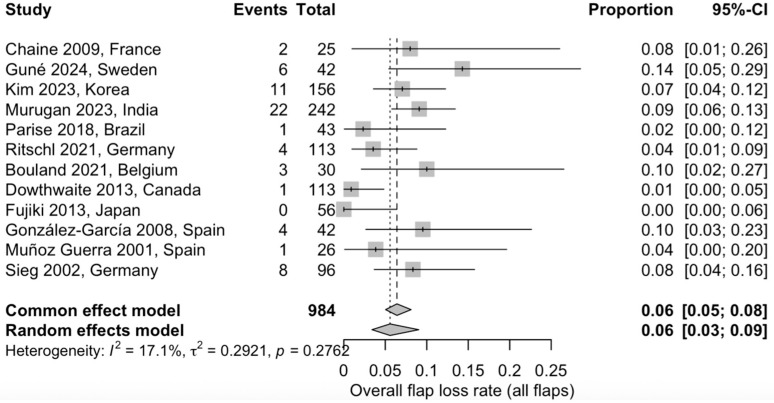
Forest plot showing the pooled proportion of total flap loss across the included studies using a random-effects model: Squares represent individual study estimates with 95% confidence intervals, while the diamond represents the pooled estimate [20,21,22,24,25,27,28,29,30,31,32,33].

A proportional meta-analysis was performed to estimate the overall incidence of total flap loss following osseous free flap reconstruction. Twelve studies, including a total of 984 flaps, were included. Using a random-effects model, the pooled flap loss rate was 6% (95% CI: 3–9%). Low statistical heterogeneity was observed across studies (I^2^ = 17.1%), indicating consistent reporting of this outcome across the included studies (Figure 3) [20,21,22,24,25,27,28,29,30,31,32,33].

#### 3.5.2. Overall Partial Flap Loss

**Figure 4:**
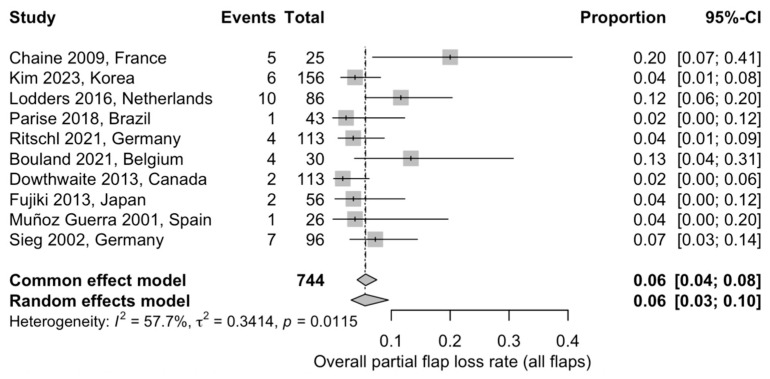
Forest plot showing the pooled proportion of partial flap loss across the included studies using a random-effects model: Squares represent individual study estimates with 95% confidence intervals, while the diamond represents the pooled estimate [20,22,23,25,27,28,29,30,32,33].

The overall incidence of partial flap loss was assessed through a proportional meta-analysis including ten studies and 744 flaps. The pooled partial flap loss rate was 6% (95% CI: 3–10%) using a random-effects model. Moderate heterogeneity was observed across studies (I^2^ = 57.7%), likely reflecting differences in outcome definitions and reporting across retrospective cohorts (Figure 4) [20,22,23,25,27,28,29,30,32,33].

#### 3.5.3. Overall Postoperative Infection

**Figure 5:**
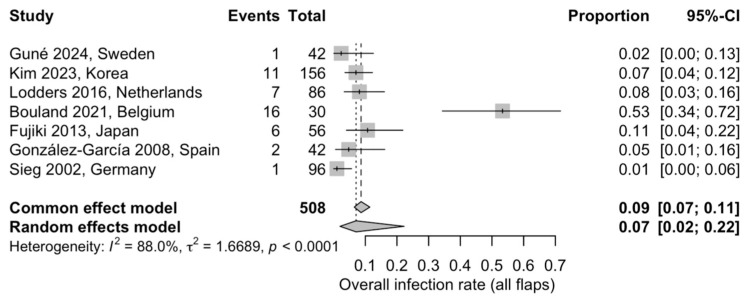
Forest plot illustrating the pooled proportion of postoperative infections across the included studies using a random-effects model: Squares represent individual study estimates with 95% confidence intervals, while the diamond represents the pooled estimate [21,22,23,28,30,31,33].

Seven studies, comprising a total of 508 flaps, were included in the meta-analysis evaluating postoperative infection. The pooled infection rate was 7% (95% CI: 2–22%) based on a random-effects model. A high degree of heterogeneity was detected (I^2^ = 88.0%), likely reflecting substantial differences in patient selection, perioperative management, and infection definitions across studies (Figure 5) [21,22,23,28,30,31,33].

#### 3.5.4. Overall Fistula Formation

**Figure 6:**
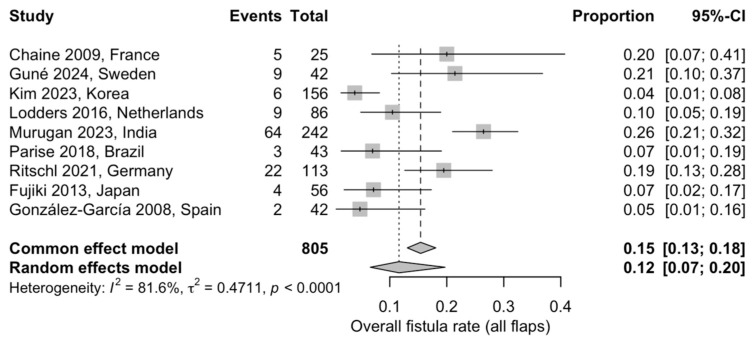
Forest plot showing the pooled proportion of postoperative fistula formation across the included studies using a random-effects model: Squares represent individual study estimates with 95% confidence intervals, while the diamond represents the pooled estimate [20,21,22,23,24,25,27,30,31].

A proportional meta-analysis of nine studies, including 805 flaps, was conducted to estimate the overall incidence of fistula formation. The pooled fistula rate was 12% (95% CI: 7–20%) using a random-effects model. Substantial heterogeneity was observed across studies (I^2^ = 81.6%), likely reflecting differences in defect site, extent of reconstruction, use of adjuvant radiotherapy, and fistula definitions across studies (Figure 6) [20,21,22,23,24,25,27,30,31].

#### 3.5.5. Overall Wound Dehiscence

**Figure 7:**
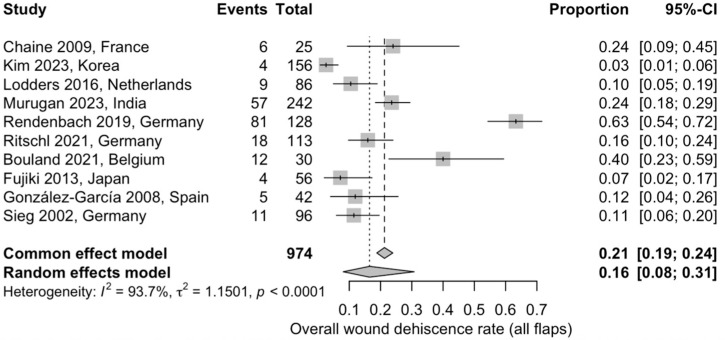
Forest plot depicting the pooled proportion of postoperative wound dehiscence across the included studies using a random-effects model: Squares represent individual study estimates with 95% confidence intervals, while the diamond represents the pooled estimate [20,22,23,24,26,27,28,30,31,33].

Ten studies, including a total of 974 flaps, were included in the meta-analysis assessing wound dehiscence. The pooled wound dehiscence rate was 16% (95% CI: 8–31%) using a random-effects model. Considerable heterogeneity was observed (I^2^ = 93.7%), likely reflecting substantial variability in defect characteristics, reconstructive techniques, adjuvant radiotherapy exposure, and definitions of wound dehiscence across studies (Figure 7) [20,22,23,24,26,27,28,30,31,33].

### 3.6. Quantitative Meta-Analysis—Flap-Related Complications

The number of studies contributing to each flap-specific meta-analysis varied according to outcome availability and completeness of data. For scapular free flaps, postoperative complications other than total flap loss were reported inconsistently across studies, precluding quantitative synthesis. Similarly, for DCIA free flaps, only two studies with small sample sizes were available, and outcome reporting was incomplete and heterogeneous. Consequently, quantitative meta-analysis was not considered methodologically appropriate. Detailed flap-related meta-analyses are reported in the Supplementary Materials.

#### 3.6.1. Fibular Free Flap

A fibular flap proportional meta-analysis was performed to evaluate postoperative complication rates. A total of 765 fibular free flaps from ten studies were included in the analysis of total flap loss, yielding a pooled flap loss rate of 6.1% (95% CI: 3.9–9.4%) using a random-effects model, with low between-study heterogeneity (I^2^ = 0.0%) (Figure S1A). The absence of statistical heterogeneity should be interpreted cautiously as low I^2^ values are expected in meta-analyses of rare outcomes with a limited number of studies. Partial flap loss was assessed in ten studies including 605 fibular free flaps, with a pooled rate of 6.6% (95% CI: 4.2–10.2%) and moderate heterogeneity (I^2^ = 39.5%) (Figure S1B). Postoperative infection was evaluated in six studies comprising 406 fibular free flaps. The pooled infection rate was 9.0% (95% CI: 2.2–30.1%) using a random-effects model, with substantial heterogeneity observed across studies (I^2^ = 88.5%) (Figure S1C). Fistula formation was reported in eight studies including 679 fibular free flaps, resulting in a pooled fistula rate of 10.4% (95% CI: 5.0–20.4%), with high heterogeneity (I^2^ = 80.3%) (Figure S1D). Wound dehiscence was assessed in ten studies including 869 fibular free flaps and showed a pooled incidence of 17.1% (95% CI: 8.3–32.0%), with considerable heterogeneity across studies (I^2^ = 93.1%) (Figure S1E). Hardware-related complications were analyzed in eight studies comprising 781 fibular free flaps, with a pooled rate of 10.4% (95% CI: 5.9–17.8%) and substantial heterogeneity (I^2^ = 81.9%) (Figure S1F). Additional postoperative complications, including hematoma, seroma, chyle leak, bone exposure, other flap-related adverse events, and revision surgery, were inconsistently reported across studies and were therefore summarized descriptively.

#### 3.6.2. Scapular Free Flap

A scapular flap-specific proportional meta-analysis was performed to evaluate flap loss rates. Five studies, including a total of 157 scapular free flaps, were included in the analysis. Using a random-effects model, the pooled flap loss rate was 5% (95% CI: 1–29%), with no statistical heterogeneity observed across studies (I^2^ = 0.0%); however, this finding should be interpreted with caution given the limited number of contributing studies and the wide confidence intervals (Figure S2) [21,22,29,30].

## 4. Discussion

Large composite defects of the mandible and maxilla remain among the most challenging issues in head and neck reconstruction. Vascularized osseous free flaps represent the current standard of care as they provide reliable bone replacement, adequate soft-tissue coverage, and the structural basis for functional and dental rehabilitation [34]. The fibular, scapular, and iliac crest free flaps are the most commonly used reconstructive options and are consistently associated with high flap survival rates [35,36,37,38,39].

Nevertheless, flap survival alone is an incomplete measure of reconstructive success. Clinically, non-vascular complications often represent the primary determinants of postoperative morbidity and may outweigh flap survival in influencing functional outcomes and patient counseling. Postoperative complications remain frequent and clinically relevant, affecting both defect and donor sites and influencing recovery, function, and quality of life.

While several narrative and systematic reviews have addressed complications following microvascular osseous reconstruction, most have reported aggregated outcomes without stratification by flap type [13,17,33,34,38,39]. Consequently, flap-specific complication patterns, which could guide the reconstructive decision-making process, remain incompletely defined. In this context, the present systematic review and meta-analysis provide a comprehensive synthesis of postoperative complications following microvascular osseous free flap reconstruction, with outcomes stratified by flap type when feasible.

Data obtained from this review showed that the pooled total flap loss rate was approximately 6%, confirming the technical reliability of contemporary microvascular techniques. This finding is consistent with previously reported series and supports flap survival as a robust indicator of microvascular success. However, the postoperative morbidity is not only reflected by flap survival alone. Non-vascular reconstruction site complications were more frequently reported, including wound dehiscence (approximately 16–17%), fistula formation (around 10–12%), postoperative infection (about 7–9%), and hardware-related complications (approximately 10%). These adverse events occurred several times more often than total or partial flap loss and accounted for the majority of reported complications. Differences between studies likely reflect clinical variability. Notably, successful flap survival does not exclude the occurrence of clinically relevant postoperative complications.

The fibular free flap was the most extensively represented reconstructive option in the available literature, allowing more robust estimation of complication rates. While the pooled flap loss rate for fibular reconstructions mirrored the overall estimate (6.1%), a distinct pattern of non-vascular morbidity emerged, with wound dehiscence (17.1%), fistula formation10.4%), hardware-related complications (10.4%), and infection (9%) representing the predominant postoperative challenges.

In contrast, evidence regarding scapular-based and deep circumflex iliac artery (DCIA) free flaps was limited. Quantitative synthesis for scapular flaps was feasible only for total flap loss, yielding a pooled estimate of approximately 5% with wide confidence intervals. Other complications were inconsistently reported, precluding reliable quantitative analysis. Similarly, data on DCIA flaps were restricted to small retrospective series with heterogeneous and incomplete reporting. As a result, complication rates for these flaps should be interpreted with caution.

Donor site morbidity was inconsistently reported across studies and could not be quantitatively synthesized. Although reported donor site complications included wound-related problems, hematoma, seroma, pain, and functional impairment, the lack of standardized reporting likely results in underestimation of the true donor site burden [21,22,23,30,31]. This represents an important limitation of the current evidence base and restricts comprehensive assessment of overall reconstructive morbidity.

Several limitations must be acknowledged. All included studies were retrospective, outcome definitions were inconsistent, and reporting was heterogeneous. Additionally, the literature search strategy was primarily based on title-level screening to maintain topic specificity, which may have led to the exclusion of potentially relevant studies not explicitly referring to osseous free flap complications in the title. Although reference lists of included studies were manually screened to mitigate this risk, incomplete study retrieval cannot be entirely excluded. Additionally, the exclusion of gray literature may have introduced a degree of publication bias as studies with negative or less prominent results are less likely to be published in peer-reviewed journals. However, this approach was chosen to ensure inclusion of studies with sufficient methodological quality and extractable quantitative data suitable for proportional meta-analysis.

Low heterogeneity observed for rare outcomes such as total flap loss should be interpreted cautiously and does not imply clinical uniformity. Conversely, the substantial heterogeneity observed for most non-vascular complications reflects significant clinical and methodological variability and limits the reliability of the pooled estimates. It was also not possible to perform a quantitative meta-analysis of donor site complications because of the lack of homogeneous data.

The substantial heterogeneity observed for several outcomes—particularly postoperative infection, fistula formation, wound dehiscence, and hardware-related complications—likely reflects marked clinical and methodological variability across the included studies. Relevant sources of heterogeneity include differences in reconstructive indication (predominantly oncologic versus mixed etiologies), defect site and extent, exposure to adjuvant radiotherapy, follow-up duration, and institutional surgical protocols, as well as inconsistent outcome definitions and reporting denominators. Although subgroup or sensitivity analyses could theoretically help identify drivers of heterogeneity, such analyses were not feasible, due to sparse and inconsistently reported data across studies and subgroups.

Notably, flap-specific analyses partially mitigated heterogeneity for selected outcomes, with low heterogeneity observed for total flap loss in fibular free flap reconstructions, supporting the robustness of flap survival estimates. In contrast, non-vascular complications remained highly heterogeneous, underscoring that pooled point estimates should be interpreted with caution and viewed as summary indicators rather than precise estimates applicable to individual patients.

Clinically, these findings highlight the importance of individualized reconstructive planning that integrates patient-specific risk factors, defect characteristics, and anticipated adjuvant treatments, rather than reliance on pooled complication rates alone. Nevertheless, awareness of flap-specific complication profiles may assist surgeons in tailoring reconstructive strategies to individual patient risk factors, provided that these results are interpreted with caution and within the appropriate clinical context, rather than being considered precise predictors of individual patient outcomes. Further studies with standardized and comprehensive reporting are needed to improve the external validity of reconstructive outcome data.

## 5. Conclusions

In conclusion, microvascular osseous free flap reconstruction is associated with high flap survival rates but a substantial burden of non-vascular postoperative complications. Flap-specific complication profiles, particularly for fibular free flaps, provide clinically meaningful information for patient counseling and reconstructive planning when interpreted within the appropriate clinical context. These findings emphasize the need to move beyond flap survival as the sole outcome measure and highlight the importance of standardized, flap-specific reporting of both recipient and donor site complications in future studies.

## Figures and Tables

**Table 1:** Features of the 14 included studies.

Author/Year/	Country	Study Design	N Cases (Sex/Age)	Flap Type	Donor Site Complications	Reconstruction Site Complications	Follow-Up
Chaine 2009 [20]	France	Retrospective observational	25 (80% M; mean 43 y)	25 FFF	FFF: - 1 skin graft required; - 1 soft-tissue contracture	FFF: - 2 total flap loss; - 5 partial flap loss; - 5 fistulas; - 6 wound dehiscence; - 1 hematoma	Mean 47 m
Guné 2024 [21]	Sweden	Retrospective observational	42 (60% M; median 63 y)	42 SFF	SFF: - 2 infections; - 2 seromas	SFF: - 6 total flap loss; - 1 infection; - 9 fistulas	Mean 49 m
Kim 2023 [22]	Korea	Retrospective observational	138 (68.1% M; mean 56 y)	114 FFF; 42 SFF	FFF: - 6 skin graft loss; - 1 dehiscence; - 2 compartment syndrome; SFF: 0	FFF: - 5 total flap loss; - 6 partial flap loss; - 10 infections; - 2 fistulas; - 4 wound dehiscence; - 4 hardware complications SFF: - 6 total flap loss; - 1 infection; - 4 fistulas; - 3 hardware complications	Mean 44 m
Lodders 2016 [23]	Netherlands	Retrospective observational	85 (45.9% M; mean 61.2 y)	86 FFF	FFF: - 1 wound infection; - 1 wound necrosis; - 1 wound dehiscence	FFF: - 10 partial flap loss; - 7 infections; - 9 fistulas; - 2 bone exposure; - 6 hardware complications; - 5 hematomas; - 2 seromas; - 3 chyle leak; - 22 revision surgeries	Mean 45 m
Murugan 2023 [24]	India	Retrospective observational	242 (78.5% M; mean 44.3 y)	242 FFF	FFF: not numerically quantified	FFF: - 22 total flap loss; - 64 fistulas; - 57 wound dehiscence; - 26 bone exposure; - 30 hardware complications	Mean 20 m
Parise 2018 [25]	Brazil	Retrospective observational	43 (51% M; mean 44 y)	43 FFF	FFF: not reported	FFF: - 1 total flap loss; - 1 partial flap loss; - 3 fistulas; - 4 hardware complications	NR
Rendenbach 2019 [26]	Germany	Retrospective observational	128 (64.1% M; mean 59.2 y)	107 FFF; 8 SFF; 13 DCIA	FFF: not reported; SFF: not reported; DCIA: not reported	FFF: - 72 wound dehiscence; - 41 bone exposure; - 33 hardware complications SFF: - 3 hardware complications	Mean 15.5 m
Ritschl 2021 [27]	Germany	Retrospective observational	113 (63.7% M; mean 59 y)	89 FFF; 24 DCIA	FFF: not reported; DCIA: not reported	FFF: - 4 total flap loss; - 4 partial flap loss; - 18 fistulas; - 16 wound dehiscence; - 6 bone exposure; - 11 hardware complications; - 10 revision surgery DCIA: - 4 fistulas; - 2 wound dehiscence; - 1 bone exposure; - 2 hardware complications	Mean 61 m
Bouland 2021 [28]	Belgium	Retrospective observational	30 (66.7% M; mean 52 y)	30 FFF	FFF: not numerically quantified	FFF: - 3 total flap loss; - 4 partial flap loss; - 16 infections; - 12 wound dehiscence	NS
Dowthwaite 2013 [29]	Canada	Retrospective observational	110 (61.8% M; mean 62 y)	58 FFF; 55 SFF	FFF: - 1 hematoma; - 5 wound dehiscence SFF: - 1 hematoma; - 3 seromas	FFF: - 1 partial flap loss; - 5 hardware complications SFF: - 1 total flap loss; - 1 partial flap loss; - 3 hardware complications; - 5 revision surgeries	NR
Fujiki 2013 [30]	Japan	Retrospective observational	56 (69.6% M; mean 63 y)	38 FFF; 18 SFF	FFF: - 13 partial skin graft loss; - 2 skin grafts at the donor site; - 3 wound infections; - 1 wound dehiscence SFF: - 1 hematoma; - 1 seroma	FFF: - 2 partial flap loss; - 5 infections; - 4 fistulas; - 2 wound dehiscence; - 2 revision surgeries SFF: - 1 infection; - 2 wound dehiscence; - 1 hematoma; - 2 seromas	1 m
González-García 2008 [31]	Spain	Retrospective observational	42 (64.3% M; mean 52 y)	42 FFF	FFF: - 3 suture dehiscence; - 2 pain; - 2 hematomas; - 1 infection; - 1 fistula	FFF: - 4 total flap loss; - 2 infections; - 2 fistulas; - 5 wound dehiscence; - 4 hardware complications; - 1 hematoma	Mean 20.5 m
Muñoz-Guerra 2001 [32]	Spain	Retrospective observational	26 (80.8% M; mean 55.2 y)	26 FFF	FFF: - 10 transitory pain; - 7 transitory peroneal sensory loss; - 10 transitory weakness of the extensor hallucis longus muscle	FFF: - 1 total flap loss; - 1 partial flap loss; 6 revision surgeries	Mean 17 m
Sieg 2002 [33]	Germany	Retrospective observational	93 (74.2% M; range 15–81 y)	96 FFF	FFF: Not numerically quantified	FFF: - 8 total flap loss; - 7 partial flap loss; - 1 infection; - 11 wound dehiscence	NS

Abbreviations: NR, not reported; NS, not specified; m, months; FFF, fibular free flap; SFF, scapular free flap; DCIA, deep circumflex iliac artery flap.

## Data Availability

The data that support the findings of this study are available from the corresponding author upon reasonable request.

## Supplementary Materials

The following supporting information can be downloaded at https://www.mdpi.com/article/10.3390/jcm15020797/s1: Figure S1: Forest plots showing pooled proportions of postoperative complications following fibular free flap reconstruction using random-effects meta-analysis; Figure S2: Forest plot illustrating the pooled proportion of total flap loss following scapular free flap reconstruction; Table S1: PRISMA 2020 Main Checklist; Table S2: PRIMSA Abstract Checklist.

## Abbreviations

DCIA flap: deep circumflex iliac artery flap (iliac crest osseous free flap); FFF: fibular free flap; LSBF: lateral scapular border flap; M/F: male/female; SFF/SFF flap: scapular free flap; SOFF: scapular osseous free flap; STFF: scapular tip free flap.
